# GYY4137, as a slow-releasing H_2_S donor, ameliorates sodium deoxycholate–induced chronic intestinal barrier injury and gut microbiota dysbiosis

**DOI:** 10.3389/fphar.2024.1476407

**Published:** 2024-10-22

**Authors:** Shaorong Pan, Han Yan, Jing Zhu, Yuanyuan Ma, Pengyuan Wang, Yucun Liu, Zeyang Chen

**Affiliations:** ^1^ Department of Gastrointestinal Surgery, Peking University First Hospital, Peking University, Beijing, China; ^2^ Animal Experiment Center, Peking University First Hospital, Peking University, Beijing, China

**Keywords:** GYY4137, sodium deoxycholate, intestinal barrier, tight junction protein, gut microbiota dysbiosis

## Abstract

**Introduction:**

Numerous studies have revealed that a long-term high-fat diet can raise intestinal deoxycholate acid concentration, which can harm intestinal mucosal barrier function in several ways. This study aims to verify the protective effect of GYY4137, as a slow-releasing H_2_S donor, on microbiome disturbance and the chronic injury of the intestinal mucosal barrier function caused by sodium deoxycholate.

**Methods:**

Caco-2 monolayer and mouse models were treated with a relatively high concentration of sodium deoxycholate (1.0 mM and 0.2%, respectively) for longer periods (32 h and 12 weeks, respectively) to understand the effects of GYY4137 on sodium deoxycholate–induced chronic intestinal barrier dysfunction and its fundamental mechanisms.

**Results:**

A relatively long period of sodium deoxycholate treatment can remarkably increase the intestinal barrier permeability, alter the distribution and expression of tight junction proteins and generate the production of pro-inflammatory cytokines (TNF-*α* and IL-1*β*) in the Caco-2 monolayers and mouse models. Moreover, it can activate the MLCK-P-MLC2 pathway in the Caco-2 monolayers, which was further confirmed using RNA sequencing. The body weight, intestinal barrier histological score, and TUNEL index of sodium deoxycholate-treated mice worsened. In addition, an induced microbiome imbalance was observed in these mice. The above variations can be reversed with the administration of GYY4137.

**Conclusion:**

This study demonstrates that GYY4137 ameliorates sodium deoxycholate–induced chronic intestinal barrier injury by restricting the MLCK-P-MLC2 pathway while elevating the expression level of tight junction proteins, anti-apoptosis and maintaining the microbiome’s homeostasis.

## 1 Introduction

Obesity has emerged as a significant global public health challenge, increasing the risk of several major non-infectious diseases and lowering the population’s average life expectancy. The proportion of people with obesity worldwide has risen from 3% to 12% among men and from 7% to 16% among women between 1975 and 2016 (Sung et al., 2019). The increased high-fat diet (HFD) intake has largely contributed to the spread of obesity (Pan et al., 2021). Obesity and long-term intake of HFD impair intestinal mucosal barrier function (IMBF) in different ways (Li and Li, 2020; Tanaka et al., 2020) and are strongly associated with the occurrence of inflammatory bowel disease (IBD) (Lee et al., 2020).

Bile acids can be divided into two categories: primary and secondary bile acids. Primary bile acids can be transformed into secondary bile acids via hydrolysis and dehydrogenating bacteria in the colon, and deoxycholate acid (DCA) is the most crucial component of secondary bile acids (Camilleri and Vijayvargiya, 2020). A long-term HFD intake can increase intestinal DCA concentration (Reddy et al., 1980; Bajor et al., 2010; Stenman et al., 2012; Beau et al., 2023; Lv et al., 2023). DCA can impair IMBF in various ways, such as dysbiosis, aberrant mucosal immune response and so on (Fuchs and Trauner, 2022). Mroz et al. (2018) discovered that DCA could restrict IMBF by inhibiting the regeneration and migration of intestinal mucosal epithelial cells. Liu et al. (2018) and Chen et al. (2019) reported that DCA or sodium deoxycholate (SDC) could hinder IMBF by affecting the expression and distribution of tight junction proteins (TJPs), Occludin and ZO-1. In addition, altering intestinal DCA concentration can lead to microbiome imbalance, resulting in several metabolic diseases, such as obesity (Wahlström et al., 2016; Ma et al., 2022; Gao et al., 2023).

In the traditional concept, hydrogen sulphide (H_2_S) is a potentially toxic gas molecule with a pungent odor. It is considered one of the prominent gas molecules causing air pollution (Wallace et al., 2017). However, further investigation deems endogenous H_2_S to be the third major gas signaling molecule after nitric oxide and carbon monoxide. H_2_S is involved in various physiological functions of the human body, such as dilating blood vessels (Barton and Meyer, 2019), anti-inflammation (Cao et al., 2020), and protecting the integrity of gastric mucosa (Magierowski et al., 2015). H_2_S can exert its protective effect on IMBF in different ways. Blackler et al. (2015), Motta et al. (2015) demonstrated that H_2_S can alleviate the dysbiosis of the gut microbiota and rebuild the intestinal mucous layer. Koike et al. (2020) discovered that H_2_S could enhance the local blood perfusion of the intestinal mucosa by facilitating angiogenesis and vasodilation to protect IMBF. H_2_S is also believed to restrict the infiltration of inflammatory cells into tissues and the production of inflammatory cytokines, thereby reducing their impairment of IMBF (Zhao et al., 2016; Wallace et al., 2017). GYY4137 is a slow-releasing H_2_S donor compound that discharges H_2_S upon hydrolysis, thereby simulating the slow production of endogenous, physiological H_2_S (de Mello et al., 2022). Previous studies have explored the protective effect of H_2_S on IMBF impairment caused by endotoxemia (Chen et al., 2016).

TJP is one of the most vital structures for maintaining IMBF, and research on the effects of GYY4137 on its ultrastructure and physiological function is still minimal. In this study, cells and animals were treated with a relatively high concentration of SDC for a relatively long time to establish the chronic injury model of IMBF and confirm the protective effect of GYY4137 on the microbiome disturbance and chronic injury of IMBF caused by SDC. The outcomes are expected to present a novel therapeutic target for preventing and treating obesity and deoxycholic acid–related intestinal barrier impairment.

## 2 Materials and methods

### 2.1 Reagents

SDC (CAS No.: 302-95-4), GYY4137 (CAS No.: 1975149-21-3), and FITC-Dextran (4,000 Da, FD-4, CAS No.: 60842-46-8) were purchased from Sigma-Aldrich (United States of America). The primary antibodies were purchased from the companies as follows: ZO-1 (source: mouse, Proteintech, United States); Occludin (source: rabbit, CST, United States of America); MLCK (source: rabbit, Proteintech, United Kingdom); MLC2 (source: rabbit, ABclonal, China); and P-MLC2 (Ser19) (source: rabbit, CST, United States). Alexa Fluor 488 conjugated goat anti-rabbit antibodies (catalogue no. A-11034, RRID AB_2576217) and Alexa Fluor 555 conjugated goat anti-mouse antibodies (catalogue no. A-21422, RRID AB_2535844) were purchased from Invitrogen (United States). TNF-*α* Mouse Uncoated ELISA Kit (CAS No.: 88-7324-88) and IL-1*β* Mouse Uncoated ELISA Kit (CAS No.: 88-7013A-88) were purchased from Thermo Fisher (United States). Human TNF-*α* ELISA Kit (CAS No.: GEH0004-96T) and Human IL-1*β* ELISA Kit (CAS No.: GEH0002-96T) were purchased from Servicebio (China).

### 2.2 Cell culture

This study used Caco-2 cells purchased from the American Type Culture Collection (ATCC, United States) between passages 28 and 34. Cells were maintained at 37°C and cultured in Dulbecco’s Modified Eagle’s Medium (DMEM) supplemented with 4.5 mg/mL glucose, 10% fetal bovine serum (FBS), 50 U/mL streptomycin and penicillin, and 25 mmol/L HEPES (4-(2-hydroxyethyl)-1-piperazineethanesulfonic acid) as described previously (Ye et al., 2011; Xu et al., 2012; Chen et al., 2016). For transwell growth, 10^5^ cells were seeded on filters with pore size measuring 0.4 μm (Corning Inc., United States). In total, 200 *μ*M of GYY4137 was added to the medium in the basolateral compartments of transwells with or without 1.0 mM of SDC in the apical compartments.

### 2.3 Transepithelial electrical resistance (TEER) measurements

Caco-2 cells were plated on the 12 well transwell systems, and the epithelial volt-ohm meter ERS-2 (Merck Millipore, United States) was used to measure any variations in TEER (Ma et al., 1999; Ma et al., 2000). The cells were cultured with different reagents as indicated when the epithelial resistance of Caco-2 monolayers reached 350–550 Ω cm^2^ about 3 weeks after confluence (Guo et al., 2013). The TEER was measured until similar values were documented three consecutive times.

### 2.4 Fluorescein isothiocyanate–dextran 4,000 Da (FD-4) flux measurements

Paracellular permeability was measured as previously reported (Schliwa, 1982; Schlegel et al., 2011). Following the indicated treatment, the monolayers were rinsed with phosphate-buffered saline (PBS) solution and then incubated with Hank’s balanced salt solution containing 1 mg/mL FD-4 in the apical compartments for 2 h. In total, 100 *μ*L of solution taken from the basolateral compartments was used to measure the FD-4 flux. The fluorescent signal was assessed using a Synergy H2 microplate reader (Biotek Instruments, United States) at an excitation of 492 nm and emission filters of 520 nm. Serial dilution was used to determine FD-4 concentration using standard curves.

### 2.5 Western blot

The total protein of Caco-2 monolayers was extracted following the method reported previously (Wang, 2012). A previously described method was applied for the total protein extraction of the proximal colon mucosa (2 cm) (Moriez et al., 2005; Chen et al., 2016). The bicinchoninic acid (BCA) method was used to quantify the concentration of proteins. Next, extracts containing equal quantities of proteins (20 *μ*g) were electrophoresed in 4%–20% polyacrylamide gel and the separated proteins were subsequently transferred to polyvinylidene difluoride (PVDF) membranes. The membrane was blocked for 1 hour for nonspecific binding (5% bovine serum albumin [BSA] in TBS-Tween 20 buffer) at room temperature and then incubated with primary antibodies (1:1,000 dilution) at 4°C overnight. Subsequently, the membrane was incubated with corresponding secondary antibodies (1:10000 dilution) for 1 hour at room temperature. The blots were developed with electrochemiluminescence (ECL) detection reagents (Merck Millipore, United States) and visualized using a Syngene GeneGenius gel imaging system (Syngene, MD).

### 2.6 Immunofluorescent of ZO-1 and occludin in Caco-2 monolayers

The cellular localization of ZO-1 and Occludin was visualized using an immunofluorescent antibody labeling technique as previously reported following indicated treatment (Wang, 2012; Chen et al., 2016). Monolayers were rinsed with PBS. Then, filters were fixed with 100% methanol at −20°C overnight and 100% acetone at −20°C for 1 min. After that, filters were blocked with 1% BSA for 1 h at room temperature and incubated with anti-mouse ZO-1 and anti-rabbit Occludin antibodies at 4°C overnight. The filters were washed with PBS, treated with Alexa fluor 488 conjugated goat anti-rabbit antibodies and Alexa fluor 555 conjugated goat anti-mouse antibodies in 1% BSA for 1 h at room temperature, and subsequently rinsed with PBS. Finally, the Prolong Gold Antifade Reagent (Molecular Probes, United States) was used, and the filters were maintained in the dark at 4°C until analysis. The fluorescence was assessed under a Fluoview 1,000 confocal microscope (Olympus, Japan).

### 2.7 Animals

**FIGURE 1:**
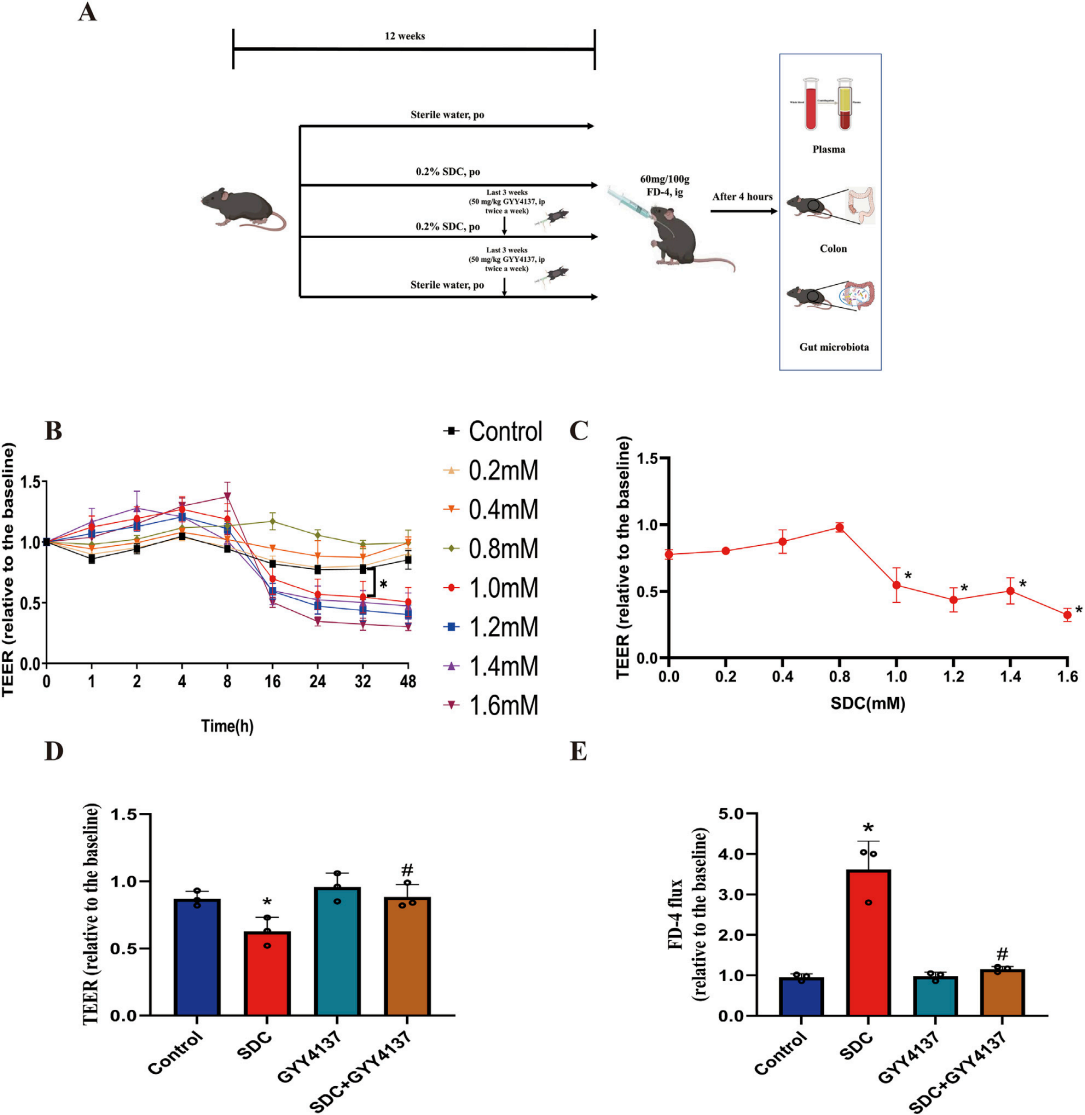
Experiment design and the effects of SDC and GYY4137 on Caco-2 monolayer barrier function. **(A)** Schematic overview of the animal experiment. **(B)** Different concentrations (0–1.6 mM) of SDC were added to the Caco-2 monolayers. 1.0 mM or greater could significantly decrease the TEER at 32 h. **(C)** Dose-response of the TEER of Caco-2 monolayers incubated with different concentrations of SDC for 32 h. Concentration ≥1.0 mM significantly reduced the TEER. **(D)** Caco-2 monolayers were pretreated with 200 *μ*M GYY4137 for 48 h and then treated with 200 *μ*M GYY4137 in the presence or absence of 1.0 mM SDC for 32 h. GYY4137 significantly attenuated the SDC-induced TEER reduction. **(E)** Caco-2 monolayers were treated as mentioned above. The SDC-induced increased FD-4 flux was significantly decreased after the addition of GYY4137. N = 3. **(B, C)** ^*^ *P* < 0.05 vs. control. Unpaired *t*-test. **(D, E)** ^*^ *P* < 0.05 vs. control. ^#^ *P* < 0.05 vs. SDC. ANOVA, Tukey’s test.

Male C57BL/6 mice (4 weeks old) were purchased from Vital River Inc. (Beijing, China) and housed under specific pathogen-free (SPF) conditions of the Laboratory at the Animal Center at the Peking University First Hospital with access to water and food (standard AIN-93G diet) *ad libitum*. All animals were acclimatized 1 week before any treatment. The mice received free access to sterile water containing 0.2% SDC for 12 weeks to construct the chronic injury model of SDC to the intestinal barrier as previously described (Liu et al., 2018). In total, twenty-four mice were randomly divided into four groups: Control, SDC, GYY4137 alone and SDC + GYY4137. They were housed in groups of six per cage. The control group mice were treated with sterile water. A total of 50 mg/kg GYY4137 was injected intraperitoneally to the mice in the GYY 4137 group twice weekly for the last 3 weeks. Mice in the SDC group were fed with sterile water containing 0.2% SDC for 12 weeks (Liu et al., 2018; Xu et al., 2021). Mice in the SDC + GYY4137 group were fed with sterile water containing 0.2% SDC for 12 weeks and injected intraperitoneally with 50 mg/kg of GYY4137 (Chen et al., 2016) twice a week for the last 3 weeks. The experimental strategy is depicted in Figure 1A. Body weight was recorded weekly until the 12th week. All mice were killed 12 h after the final treatment. All experimental procedures were approved by the Institutional Animal Care and Use Committee of Peking University First Hospital, Beijing, P. R. China (No. J2022096).

### 2.8 Measurement of intestinal permeability

The mice were gavaged with FD-4 (60 mg/100 g body weight) 4 h before killing at the end of 12 weeks. Mice were fasted for 10 h before gavage and the gavage was conducted at 8 a.m. Blood was taken from the inner canthus and centrifuged (15 min, 3,000 rpm, 4°C) to collect the plasma. The fluorescent signal was measured using a Synergy H2 microplate reader (Biotek Instruments, United States) at an excitation of 492 nm and emission filters of 520 nm. The concentration of plasma FD-4 was calculated using the standard curve.

### 2.9 Histological assessment

The proximal colons of mice in different groups were excised and embedded in paraffin. Sections (4 μm) were sliced and stained with hematoxylin and eosin (H&E). The images were obtained using a Zeiss Image light microscope (magnification: 20×; Carl Zeiss AG, Germany). The degree of mucosal damage was graded according to a previously described study (Wang Y. et al., 2019). Score 0, normal histological findings. Score 1, mucosa: villus blunting, loss of crypt architecture, sparse inflammatory cell infiltration, vacuolization, and edema; muscle layer: normal. Score 2, mucosa: villus blunting with fattened and vacuolated cells, crypt necrosis, intense inflammatory cell infiltration, vacuolization, and edema; muscle layer: normal. Score 3, mucosa: villus blunting with fattened and vacuolated cells, crypt necrosis, intense inflammatory cell infiltration, vacuolization, and edema.

### 2.10 Immunofluorescence of ZO-1 and occludin in proximal colon sections

Proximal colons were frozen in optimum cutting temperature compound (OCT) at −80°C. Sections (4 *μ*m) were cut, and immunofluorescence was conducted, as reported previously (Karczewski et al., 2010). The fluorescence was observed using a Fluoview 1,000 confocal microscope (Olympus, Japan).

### 2.11 TUNEL analysis

Proximal colons (2 cm) were obtained, paraffin-embed and sliced into sections measuring 4 *μ*m. The TUNEL analysis was conducted using the *In Situ* Cell Death Detection Kit (POD) according to the manufacturer’s instructions (Roche, Germany). A Zeiss Image light microscope was used to collect the images. The number of total and apoptotic cells per field in each slide was calculated. Four high-powered fields and a minimum of 500 cells were calculated per slide. The apoptosis index (AI) was counted following the formula: $\text{AI}=\left(\text{the number of apoptotic cells}\right)\;/\;\left(\text{the number of total cells}\right)\times 100$ .

### 2.12 Measurement of TNF-*α* and IL-1*β*

The concentrations of TNF-*α* and IL-1*β* in the mucosa of the proximal colon of mice and cell supernatant from the basolateral compartments of transwells in each group were measured using a Mouse Uncoated ELISA Kit (Thermo Fisher, United States) and the Human ELISA Kit (Servicebio, China) according to the manufacturer’s recommendations.

### 2.13 16S rRNA amplicon sequencing

The total genomic DNA was extracted from the cecum contents of mice using the TGuide S96 Magnetic Stool DNA Kit (Tiangen Biotech (Beijing) Co., Ltd.) according to the manufacturer’s instructions. The hypervariable region V3-V4 of the bacterial 16S rRNA gene was amplified with primer pairs 338F: 5′-ACT​CCT​ACG​GGA​GGC​AGC​A-3′ and 806R: 5′-GGACTACHVGGGTWTCTAAT-3’. The PCR products were checked on agarose gel and purified using the Omega DNA purification kit (Omega Inc., Norcross, GA, United States). The purified PCR products were collected and the paired ends (2 × 250 bp) were performed on the Illumina Novaseq 6,000 platform. Clean reads were then conducted on feature classification to output ASVs (amplicon sequence variants) by dada2 (Callahan et al., 2016). The ASVs counts less than 2 in all samples were filtered. Taxonomy annotation of the ASVs was performed based on the Naive Bayes classifier in QIIME2 (Bolyen et al., 2019) using the SILVA database (Quast et al., 2013) (release 138.1) with a confidence threshold of 70%. The alpha diversity was calculated and displayed using the QIIME2 and R software. The beta diversity was determined using QIIME to evaluate the degree of similarity of microbial communities from different samples. The principal coordinate analysis (PCoA) and nonmetric multidimensional scaling (NMDS) were applied to analyze the beta diversity. Furthermore, the Linear Discriminant Analysis (LDA) effect size (LEfSe (Segata et al., 2011)) was used to test the significant taxonomic difference among groups. A logarithmic LDA score of 4.0 was set as the threshold for discriminative features.

### 2.14 mRNA sequencing

The RNA from the established chronic injury model of SDC was extracted to the Caco-2 monolayer, and cDNA libraries were constructed using PCR. The qualified libraries were sequenced using the Illumina platform. The reads count for each gene in each sample was counted using HTSeq v0.6.0, and the FPKM (Fragments Per Kilobase Million Mapped Reads) was then calculated to estimate the expression level of genes in each sample. The “limma” package was used to identify differentially expressed genes between control groups and SDC-treated groups. Adjust *P*-value < 0.05 and |log2 fold change (FC)| > 1.5 were set as the cut-off values. The volcano plots were generated using the “ggplot2” R package to visualize the differentially expressed genes. The potential biological mechanism and function enrichment of the differentially expressed genes were analyzed using Gene Ontology (GO) and Kyoto Encyclopedia of Genes and Genomes (KEGG) via R packages as follows: “clusterProfiler”, “org.Hs.eg.db”, “enrichplot”, “ggplot2”, “circlize” and “GOplot”.

### 2.15 Statistical analysis

The results are defined as mean ± standard error of the mean (SEM) and analyzed using the *t*-test for unpaired data and one-way analysis of variance (ANOVA) to compare the groups followed by a Tukey’s test for *post hoc* analysis whenever required by GraphPad Prism 9.0 software. A *P*-value < 0.05 was regarded as statistically significant. ^*^ *P* < 0.05 vs. control. ^#^ *P* < 0.05 vs. SDC.

## 3 Results

### 3.1 The effects of SDC on the TEER of Caco-2 monolayer

Different concentrations (0–1.6 mM) of SDC were used to assess the ability of barrier disruption under various incubated times to develop the chronic damage model of SDC for the monolayer. At 1.0 mM or higher, there is a considerable decrease in the TEER compared with the control group for a relatively long time, 32 h (Figure 1B). After adding various concentrations of SDC to the apical compartments for 32 h, we found 1.0 mM was assessed as the minimum concentration to generate a significant decline in the TEER of the monolayer (Figure 1C).

### 3.2 GYY4137 preserved the barrier function of the Caco-2 monolayer from the damage induced by SDC

In total, 1.0 mM SDC significantly destroys the monolayer barrier function, characterised by decreasing TEER and increasing FD-4 flux following 32 h treatment. For exploring the protective effects of GYY4137 on the SDC-induced injuries of barrier function, the monolayer was pretreated with 200 *μ*M GYY4137 for 48 h as previously reported (Chen et al., 2019), followed by co-treatment with 200 *μ*M GYY4137 and 1.0 mM SDC for 32 h. This treatment greatly ameliorated the monolayer barrier damage induced by SDC (Figures 1D, E).

### 3.3 The differentially expressed genes, GO and KEGG analysis between control and SDC-treated groups

**FIGURE 2:**
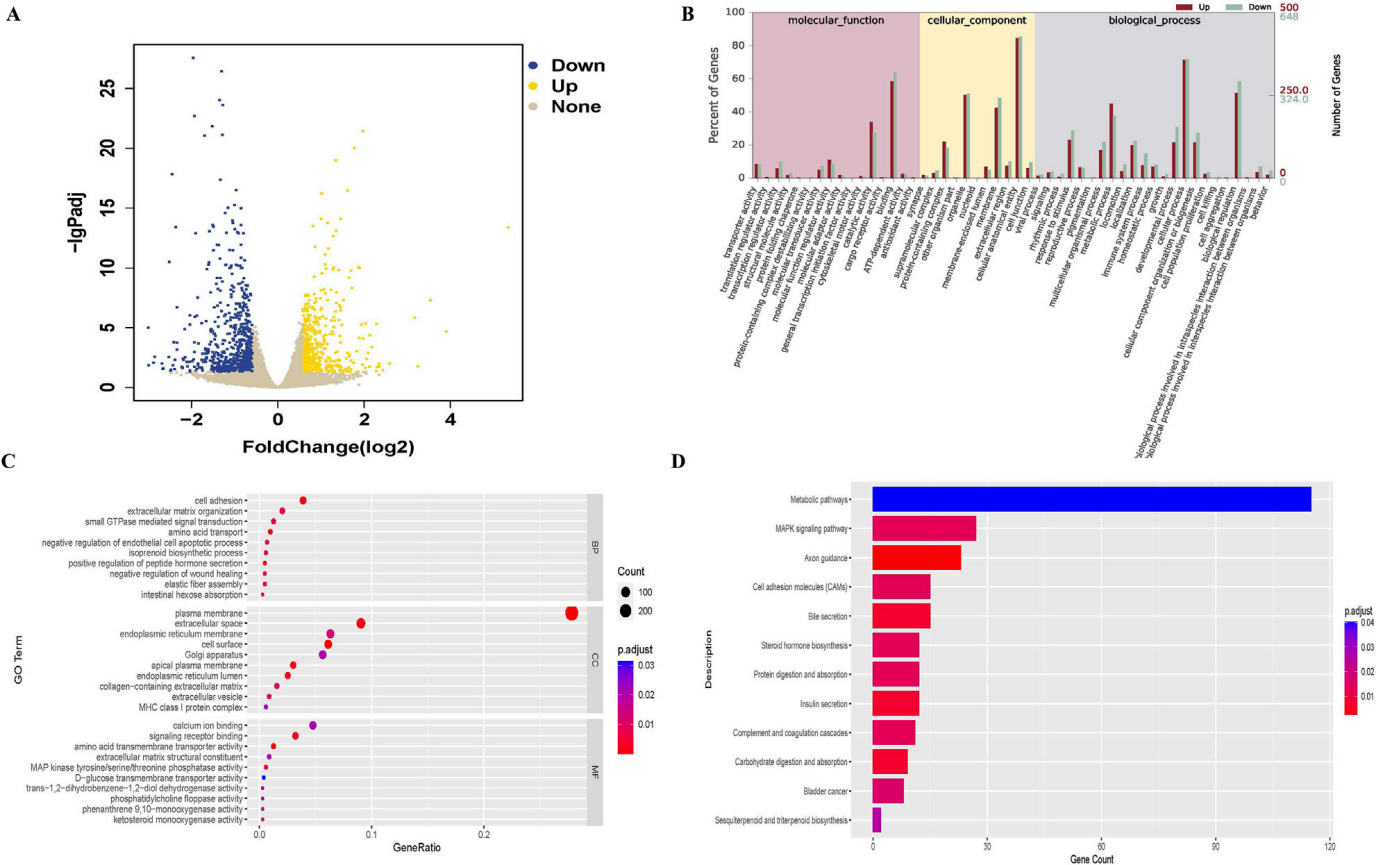
Differentially expressed genes between control (n = 3) and SDC-treated (n = 3) groups and GO, KEGG analysis. **(A)** Volcano plot of differentially expressed genes. **(B, C)** GO analysis of differentially expressed genes. **(D)** KEGG analysis of differentially expressed genes.

The genes expressions between the control and SDC-treated groups were compared, and 1,149 differentially expressed genes were identified, including 500 upregulated and 649 downregulated genes in the SDC-treated group (Figure 2A). The GO and KEGG enrichment analyses were conducted to understand the functions of any differentially expressed genes. The differentially expressed genes were primarily related to the biological processes of response to stimulus, metabolic process and cell adhesion, cellular component of the plasma membrane and extracellular space, the molecular function of calcium ion binding and signalling receptor binding according to the GO enrichment analysis (Figures 2B, C). The KEGG enrichment analysis revealed that the differentially expressed genes involved metabolic and MAPK signaling pathways (Figure 2D).

### 3.4 The effect of GYY4137 and SDC on tight junctions (TJs)

**FIGURE 3:**
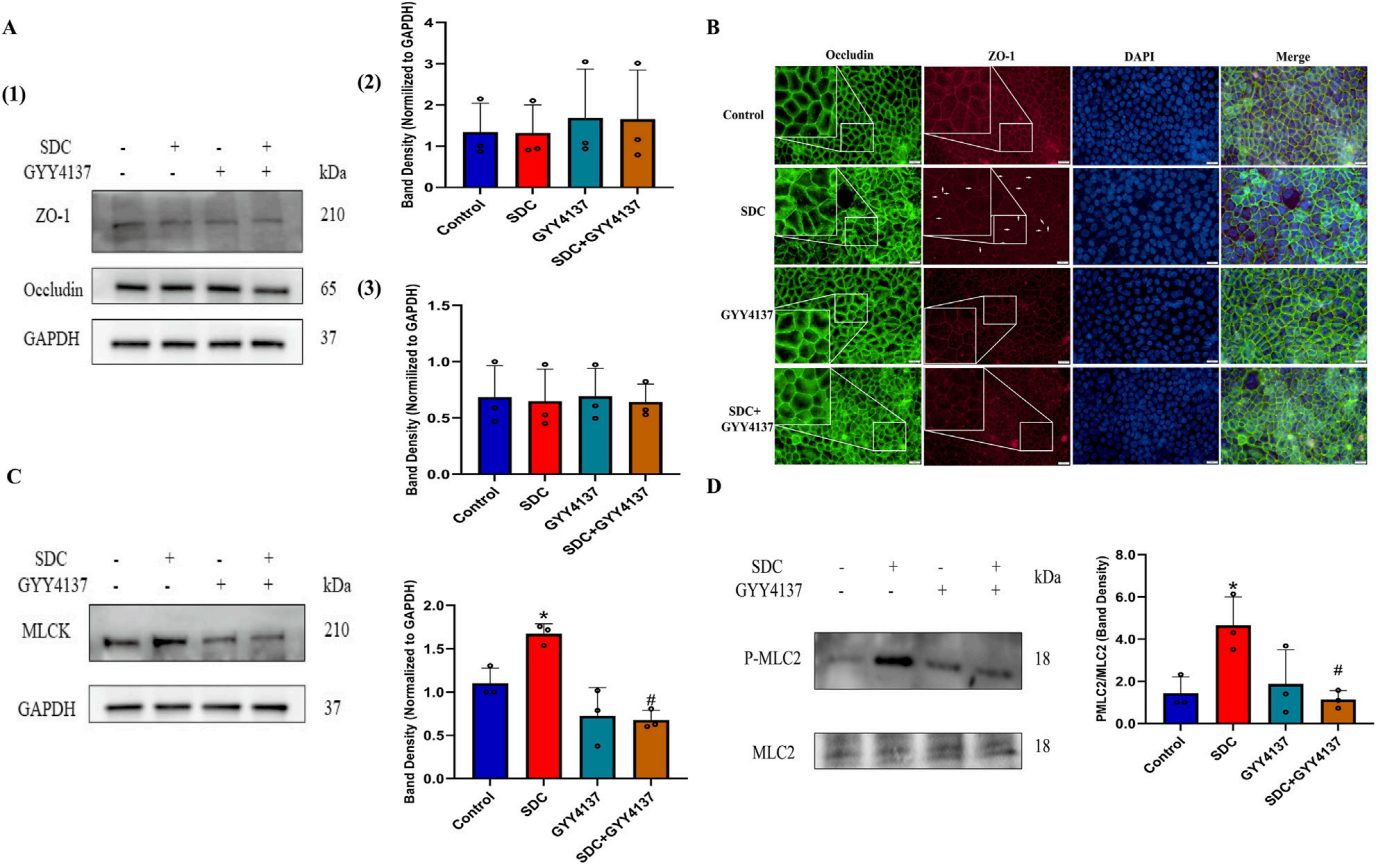
The effects of SDC and GYY4137 on TJ expression and localization and the status of MLCK-P-MLC2 signalling pathway in Caco-2 monolayers. Caco-2 monolayers were pretreated with 200 *μ*M GYY4137 for 48 h and then treated with 200 *μ*M GYY4137 in the presence or absence of 1.0 mM SDC for 32 h. The total protein of monolayers was collected after treatment for Western blot. **(A)** GYY4137 or exposure of 1.0 mM SDC for 32 h had no significant effect on the expression level of TJ proteins. **(B)** Occludin and ZO-1 were stained by immunofluorescence. GYY4137 ameliorated the altered localization of TJs caused by SDC. **(C, D)** GYY4137 significantly inhibited the increased expression of MLCK and the increased phosphorylation level of MLC2 induced by SDC. N = 3. ^*^ *P* < 0.05 vs. control. ^#^ *P* < 0.05 vs. SDC. ANOVA, Tukey’s test.

Decreased expression or altered localization of TJ proteins can hinder intestinal epithelial barrier function (Chen et al., 2016). GYY4137 or exposure of 1.0 mM SDC for 32 h exhibits no significant effect on the expression level of TJ proteins (Figure 3A). Immunofluorescences of ZO-1 and Occludin were assessed to demonstrate whether SDC could change TJ distribution. The immunofluorescences of ZO-1 and Occludin were present as smooth edges and typical chicken wire in normal conditions (Chen et al., 2016). Nevertheless, the edge of the cells appeared jagged (shown as white arrows in Figure 3B; Supplementary Figure S1), and the network structure of TJs appeared to be irregular and discontinuous when treated with 1.0 mM SDC for 32 h, indicating the abnormal distribution of TJs caused by SDC. Pretreatment and co-treatment with GYY4137 remarkably attenuated the changes induced by SDC (Figure 3B; Supplementary Figure S1).

### 3.5 GYY4137 attenuated SDC-induced activation of the MLCK-P-MLC2 signaling pathway

The MLCK-P-MLC2 signaling pathway plays a pivotal role in the regulation of TJs localization, according to previous studies (Shen et al., 2006; Chen et al., 2016). The Western blot results indicate that the increased expression of MLCK and phosphorylation level of MLC2 caused by SDC is significantly inhibited by pretreatment and cotreatment with GYY4137 (Figures 3C, D).

### 3.6 GYY4137 attenuated the increased levels of TNF-α and IL-1β

**FIGURE 4:**
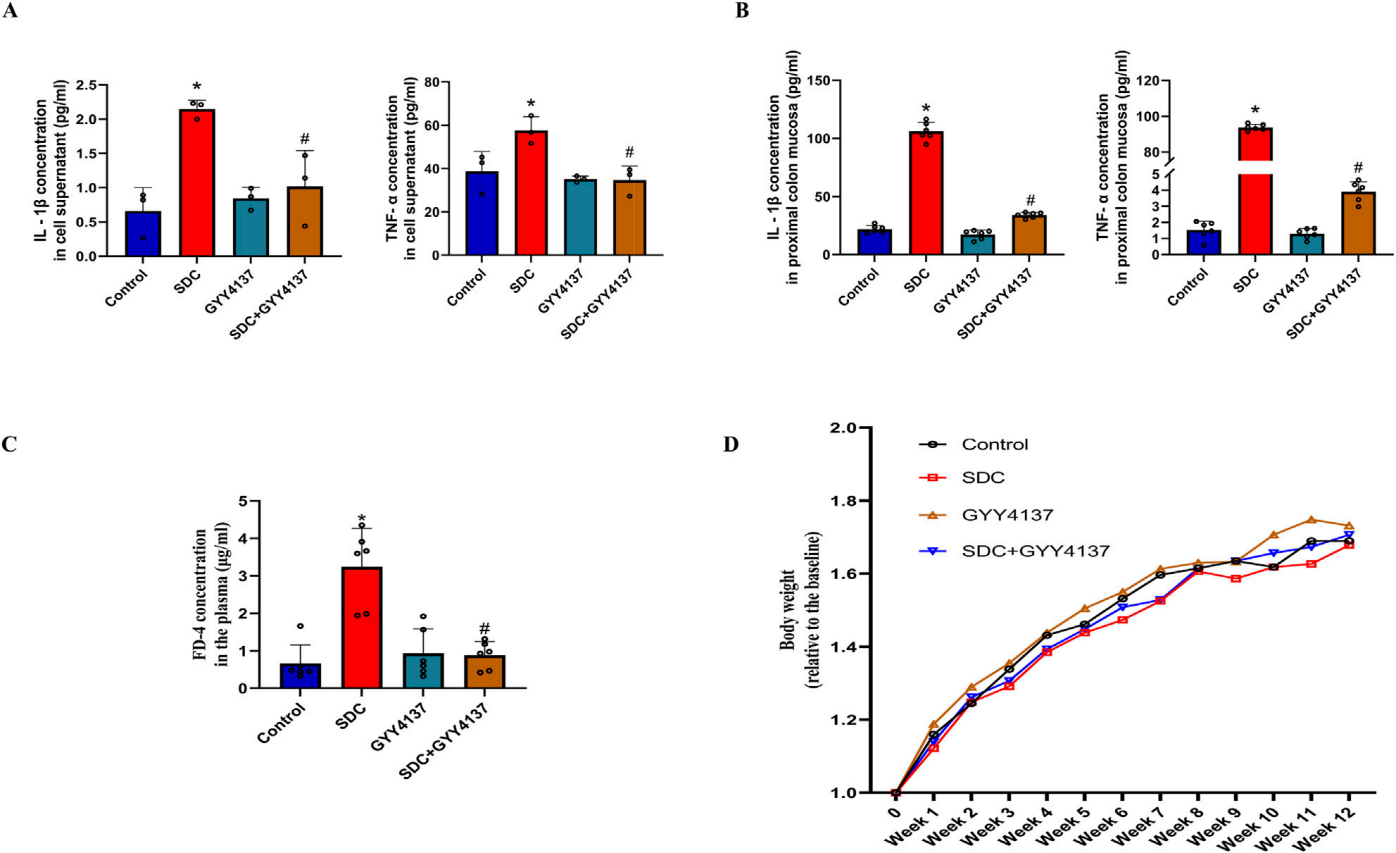
The effects of SDC and GYY4137 on the level of TNF-*α* and IL-1*β* in Caco-2 monolayers and mice, and their effects on the barrier function and body weight of mice. GYY4137 significantly decreased the increased TNF-*α* and IL-1*β* in the cell supernatant (n = 3) **(A)** and mucosa of the proximal colon of mice (n = 6) **(B)** treated with SDC. **(C)** GYY4137 significantly ameliorated the SDC-induced intestinal barrier injury featured by increased FD-4 flux in mice. N = 6. **(D)** The effects of GYY4137 and SDC on the body weight of mice. N = 6. ^*^ *P* < 0.05 vs. control. ^#^ *P* < 0.05 vs. SDC. ANOVA, Tukey’s test.

The increased levels of TNF-α and IL-1β are significantly attenuated at approximately 40% and 53%, respectively, compared with the Caco-2 monolayer treated with SDC (Figure 4A). They are also significantly reduced by around 96% and 68% in the mucosa of the proximal colon of mice treated with GYY4137 compared to mice fed with SDC alone (Figure 4B).

### 3.7 GYY4137 protected the intestinal barrier function in mice fed with SDC

The FD-4 plasma concentration in mice treated with SDC greatly increased compared to that of the control group. It was markedly decreased by co-treatment with GYY4137 (Figure 4C). These results demonstrate that GYY4137 protects the intestinal barrier function from the damage induced by SDC in mice.

### 3.8 The effects of GYY4137 and SDC on the body weight of mice

There is no significant difference in the body weight of mice between the different groups. Nevertheless, mice fed with SDC have the lowest mean body weight and the mean body weight of SDC-treated mice increased upon administering GYY4137 during the last 3 weeks (Figure 4D).

### 3.9 GYY4137 improved the histological status in mice treated with SDC

**FIGURE 5:**
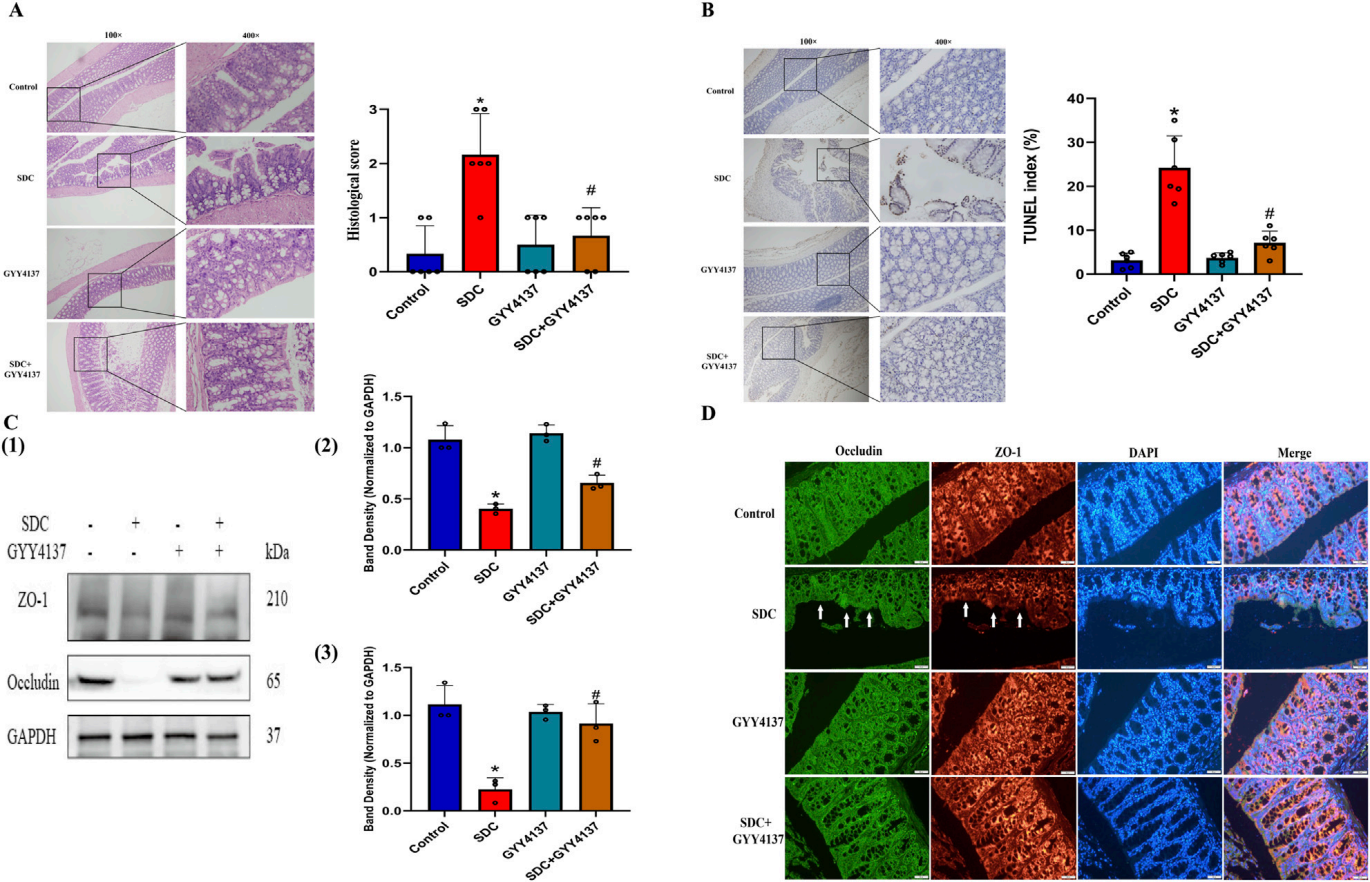
The effects of GYY4137 on the histological score, the level of apoptosis in the proximal colon epithelium and the expression of TJ proteins in mice treated with SDC. **(A)** GYY4137 remarkably attenuated the mucosal damage and increased histological score in mice treated with SDC. N = 6. **(B)** Representative TUNEL images for cell apoptosis (brown signals) and GYY4137 significantly decreased the increased level of apoptosis in the colon epithelium in mice fed with SDC. N = 6. **(C)** GYY4137 significantly ameliorated the decreased expression of ZO-1 and Occludin in the proximal colon of mice treated with SDC. N = 3. **(D)** Occludin and ZO-1 were stained by immunofluorescence. GYY4137 inhibited their decreased staining intensity and the damaged mucosal integrity in mice treated with SDC (White arrows were added to direct against distinct changes). ^*^ *P* < 0.05 vs. control. ^#^ *P* < 0.05 vs. SDC. ANOVA, Tukey’s test.

The histological injury of colon epithelium in mice treated with SDC is highlighted by villus stunting, deciduous epithelial cells, crypt disruption, and mucosal abscission, which was alleviated by cotreatment with GYY4137 (Figure 5A; Supplementary Figure S2).

### 3.10 GYY4137 preserved the proximal colon epithelial cells in mice treated with SDC from apoptosis

The increased TUNEL index in mice treated with SDC decreased considerably by approximately 71% on cotreatment with GYY4137 (Figure 5B; Supplementary Figure S3), indicating that GYY4137 might exert its function by inhibiting apoptosis.

### 3.11 The effect of GYY4137 on the status of TJ proteins in mice

The expression level of TJ proteins is remarkably decreased in the proximal colon mucosa of mice after exposure to SDC for 12 weeks. Meanwhile, GYY4137 notably attenuates the reduced expression level of TJ proteins (Figure 5C). Moreover, GYY4137 restricts the decreased staining intensity and mucosal destruction in mice caused by SDC according to immunofluorescence results (Figure 5D; Supplementary Figure S4), which was consistent with the findings of Western blot and H&E staining.

### 3.12 GYY4137 remodelled the gut microbiota in mice treated with SDC

**FIGURE 6:**
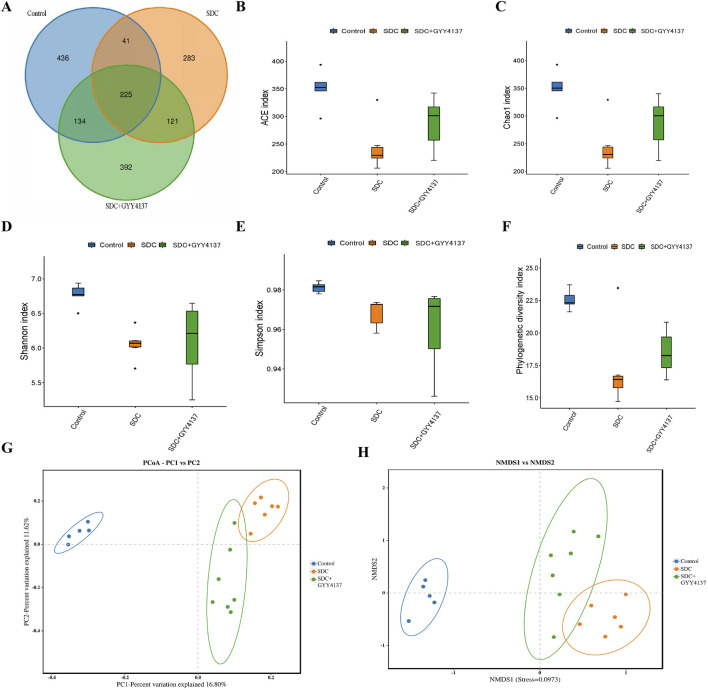
Effects of GYY4137 on gut microbiota composition in SDC-fed mice. **(A)** Venn diagram of bacterial species. **(B)** ACE index. **(C)** Chao1 index. **(D)** Shannon index. **(E)** Simpson index. **(F)** Phylogenetic diversity index. **(G)** principal coordinate analysis (PCoA) and non-metric multidimensional scaling (NMDS) analysis **(H)** based on the binary jaccard. Control: control group (n = 5), SDC: SDC group (n = 6), SDC + GYY4137: SDC + GYY4137 group (n = 7).

A high-throughput sequencing of 16S rRNA using cecum contents was conducted to compare the gut microbiota composition that underwent different interventions. The three groups share 225 ASVs, as shown in a Venn diagram (Figure 6A), and each group has its own ASVs. According to ace and chao1 indices, SDC significantly decreases the species richness of microbiota (*P* < 0.05), whereas GYY4137 seemed to offset this trend albeit without statistical significance (Figures 6B, C). The same results exhibited in Shannon, Simpson and Phylogenetic diversity indices, reflecting gut microbiota diversity (Figures 6D–F). PCoA and NMDS analysis based on the binary Jaccard demonstrated a separation between control and SDC groups, which revealed that the structure of the gut microbes changed considerably on treatment with SDC. The GYY4137 treated groups were observed to be separated from the SDC groups (Figures 6G, H).

**FIGURE 7:**
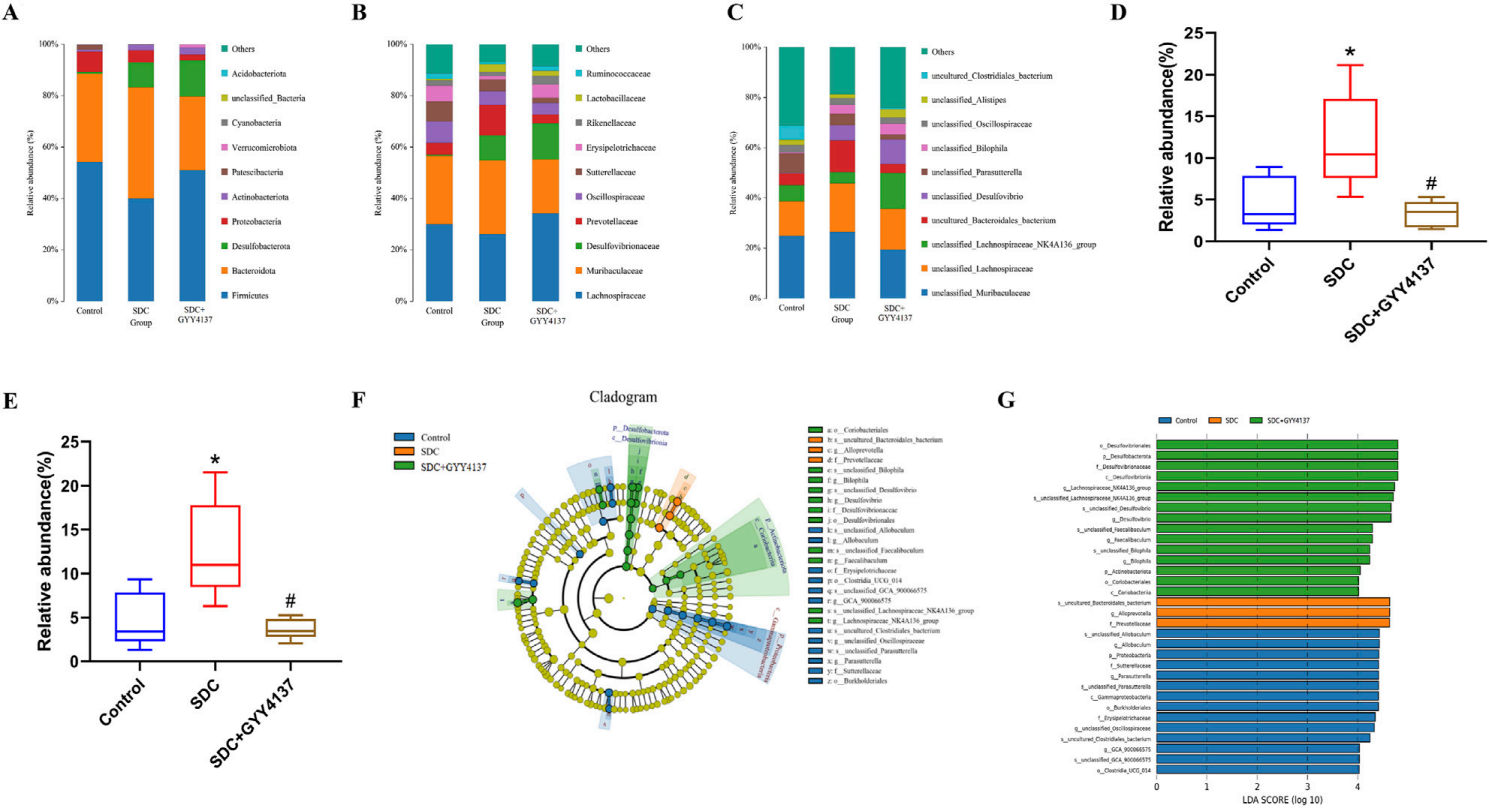
Effect of GYY4137 on microbiota composition and relative abundance in SDC-treated mice. **(A)** At the phylum level. **(B)** At the family level. **(C)** At the species level. Relative abundance of Prevotellaceae **(D)** and Bacteroidales_bacterium **(E)**. **(F)** Cladogram of LEfSe analysis. **(G)** LDA scores of LEfSe analysis. Control: control group (n = 5), SDC: SDC group (n = 6), SDC + GYY4137: SDC + GYY4137 group (n = 7). ^*^ *P* < 0.05 vs. control. ^#^ *P* < 0.05 vs. SDC. ANOVA, Tukey’s test.

Most of the phyla in the gut microbiota of all groups are *Firmicutes* and *Bacteroidota* on the phylum level without significant differences in their relative abundance among groups (Figure 7A). However, the trend in their relative abundance between the control and SDC-treated groups was consistent with previous studies (Xu et al., 2021). The relative abundance of *Prevotellaceae* significantly increased in the SDC-treated group compared with the control group at the family level, and GYY4137 administration could remarkably reverse this change (*P* < 0.05) (Figures 7B, D). In addition, SDC-treated mice depict a substantial increase in the relative abundance of *Bacteroidales_bacterium* at the species level, which was reversed by GYY4137 (*P* < 0.05) (Figures 7C, E).

The LEfSe analysis (LDA effect size) was conducted to further explore the groups’ dominant bacteria (Figures 7F, G). The results reveal that the SDC-treated group has a considerably higher abundance of the species *Bacteroidales_bacterium*, the genus *Alloprevotella* and the family *Prevotellaceae* (*P* < 0.05). The dominant genera of the SDC and GYY4137-treated group are *Lachnospiraceae_NK4A136_group*, *Desulfovibrio* and *Faecalibaculum*.

## 4 Discussion

The intestinal barrier is primarily formed by TJs of epithelial cells, which play an essential role in maintaining homeostasis of the internal environment. TJs can resist the transepithelial dissemination of pathogens and harmful antigens comprising transmembrane proteins (occludin, claudins) and accessory proteins (zonula occludens) (Mei et al., 2022). The impairment of IMBF can occur in many diseases, such as IBD and obesity. Obesity is often accompanied by a long-term HFD, increasing the incidence of IBD in this population and the concentration of secondary bile acids in the gut (Suzuki and Hara, 2010; Stenman et al., 2012; Stenman et al., 2013). As the main component of secondary bile acids, DCA has been demonstrated to influence IMBF by affecting TJ expression and distribution (Sun et al., 2004; Edelstein et al., 2011; Murakami et al., 2016; Chen et al., 2019). Therefore, the intestinal barrier can be used as a target to search for potential therapeutic reagents for IMBF impairment caused by high concentrations of DCA in people with long-term HFD.

As a novel H_2_S donor, GYY4137 can release H_2_S stably over a more extended period of time under physiological conditions than NaHS (Rose et al., 2015). GYY4137 can be anti-inflammatory and anti-apoptotic in various tissues (Zanardo et al., 2006; Meng et al., 2015). Besides, GYY4137 protects against endotoxemia and acute intestinal barrier function impairment caused by high SDC by regulating TJ expression and distribution (Chen et al., 2016; Chen et al., 2019). For a population with a long-term HFD, the DCA in the gut is in a relatively high concentration for a long time. Therefore, this study established a chronic model of intestinal barrier injury by treating SDC with a relatively high concentration for a relatively long time to explore the protective effect of GYY4137 on it, which is closer to the actual clinical physiological state.

This study explores the chronic impairment of IMBF by SDC via Caco-2 monolayers *in vitro*. Previous studies have revealed that SDC treatment for a short term can cause acute impairment to IMBF through direct destructive effects and inducing apoptosis (Edelstein et al., 2011; Tan et al., 2013; Chen et al., 2019). The Caco-2 monolayers must be treated with a relatively high concentration of SDC for a relatively long time to establish a chronic injury model of IMBF and maintain maximum cell viability. The conditions of 1.0 mM and 32 h could meet this requirement; therefore, this concentration and time were used for subsequent experiments. However, this condition did not alter the expression level of TJs in Caco-2 monolayers. The immunofluorescence indicated that the distribution of TJs changed under this condition and GYY4137 could resist this change. Some cytokines can regulate the opening of TJs by increasing the phosphorylation of MLC (Moriez et al., 2005). Therefore, the concentrations of TNF-*α* and IL-1*β* in the cell monolayer culture medium were investigated under corresponding treatment conditions, and the data demonstrated that these two cytokines were significantly increased in the SDC- treated group, and GYY4137 could antagonize such changes. In addition, the MLCK-P-MLC2 signalling pathway regulates barrier function by altering TJ distribution. An increase in the MLCK expression level can induce MLC2 phosphorylation. When the MLC2 phosphorylation level increases, actin-myosin filaments can contract, resulting in changes in ZO-1 and Occludin distribution consistent with this current research (Moriez et al., 2005; Shen et al., 2006), which demonstrated that SDC could cause the activation of this pathway and eventually lead to the dysfunction of the Caco-2 monolayer barrier function, whereas GYY4137 treatment could significantly inhibit the activation of this signalling pathway. Shen L et al. demonstrated that TJ ultrastructure was not significantly changed by MLCK expression in fully differentiated epithelial monolayers (Shen et al., 2006). However, activating this signaling pathway considerably changed TJs’ ultrastructure in mice with endotoxemia (Chen et al., 2016). The discrepancy between the two studies may be attributed to the disparity in the experimental models, warranting further investigation. Simultaneously, RNA sequencing of the IMBF chronic injury Caco-2 monolayer was performed, highlighting that the differential genes were mainly enriched in the biological components related to cell membranes, molecular functions of calcium ion binding, biological processes related to metabolic processes and stimulus response in GO analysis, while the differential genes were mainly enriched in the metabolic and MAPK signalling pathways in KEGG analysis. The activation of the MAPK pathway can increase the expression of MLCK and lead to changes in the downstream pathway (Liu et al., 2022), consistent with the results from this current study. In addition, actin-myosin filament contraction induced by MLC2 phosphorylation is also related to calcium ion flow (Tiruppathi et al., 2002; Makieva et al., 2016; Guo Z. et al., 2023), which is consistent with the GO analysis results.

The mouse model was established by freely feeding water containing 0.2%SDC for 3 months to validate the study results further. As one of the classic symptoms of IMBF impairment, GYY4137 inhibited SDC-induced weight loss to some extent; however, the difference was not statistically significant. There was no significant weight loss in the SDC treatment group, similar to previous research (Xu et al., 2021), which may be because intestinal inflammation under this condition could not cause obvious gastrointestinal symptoms or intestinal ulcers, which could not affect the daily eating behavior of mice. Furthermore, a longer treatment period with this concentration may result in more significant changes in the weight of mice, and determining this time will be one of future research objectives for this study’s authors. The intestinal barrier permeability was evaluated by measuring the FD-4 concentration of plasma, and the results showed that GYY4137 could protect against the IMBF impairment caused by SDC. Histological evaluation of colonic mucosal epithelium suggested that GYY4137 could facilitate the repair of abnormal histopathological features induced by SDC. The increased apoptosis level of intestinal epithelial cells is considered one of the crucial potential mechanisms of IMBF impairment (Coopersmith et al., 2003; Williams et al., 2013). In this study, the TUNEL analysis indicated that GYY4137 significantly reduced the SDC-caused increased apoptosis level of colonic epithelial cells. The breakdown of the intestinal barrier usually causes the accumulation of various inflammatory cells in the colon tissue, resulting in elevated levels of cytokines involved in the inflammatory response (Mei et al., 2022). The pro-inflammatory cytokines were significantly upregulated in the colonic tissues of SDC-treated mice as previously reported (Xu et al., 2021). GYY4137 considerably inhibited this trend. In addition, GYY4137 could alleviate the decrease of TJ expression levels caused by SDC. The nonconformity in the change in the tight junction protein expression between cell and animal study may be attributed to the use of different research models.

IMBF also has an essential correlation with gut microbiota. The dysbiosis of the microbiome can cause the impairment of IMBF and facilitate bacterial translocation. Intestinal pathogens can damage the barrier function by affecting the TJs of the mucosal epithelium (Wang J. et al., 2019). Therefore, future studies can target IMBF and focus on gut microbiota. Previous studies have revealed that bile acids can cause the dysregulation of the gut microbiota, which may induce IMBF impairment, resulting in intestinal inflammation (Wahlström et al., 2016; Xu et al., 2021). This study showed similar results. SDC tends to decrease microbiome’s α and β diversity, while GYY4137 could reverse this trend. The *Firmicutes*/*Bacteroidetes* ratio of the SDC group was ruduced compared with the control group. The study has revealed that a reduction in this ratio was related to weight loss (Petito-da-Silva et al., 2023). In addition, the relative abundance of *Bacteroidales_bacterium*, *Alloprevotella* and *Prevotellaceae* in the SDC-treated group had increased greatly. At the same time, the dominant bacteria changed after applying GYY4137, wherein *Lachnospiraceae_NK4A136_group*, *Desulfovibrio* and *Faecalibaculum* had shown an increase in relative abundance. Previous studies have demonstrated that *Bacteroidales_bacterium* was pathogenic (Wang et al., 2022) and found to be negatively associated with thiamine metabolism, an essential human nutrient (Liu et al., 2023). *Alloprevotella* can lead to diarrhea by promoting the formation of short-chain fatty acids (SCFAs) and inflammation, while excessive SCFAs and long-term, low-grade inflammation can result in diarrhea (Tang et al., 2023). *Alloprevotella* has also been reported to significantly increase in an irritable bowel syndrome (IBS)-like cohort (Lin et al., 2023). Diarrhea and IBS are closely related to the impairment of IMBF. Moreover, the level of *Alloprevotella* was decreased, thus alleviating intestinal inflammation and reducing intestinal diseases and potentially pathogenic bacteria following the addition of antioxidants (Gao et al., 2022). An increase in *Prevotellaceae* has been reported in patients with IBD (Darnaud et al., 2018). *Prevotellaceae* may actively degrade mucous oligosaccharides by producing elevated sulfatases in intestinal biopsies of patients with IBD, thus impairing IMBF (Elinav et al., 2011). Moreover, a high level of *Prevotellaceae* was not only associated with obesity, but also was significantly associated with insulin resistance, hypertension, periodontal disease and non-alcoholic fatty liver disease (Zhang et al., 2009; Elinav et al., 2011; Serena et al., 2018; Huang et al., 2022; Wei et al., 2024). *Lachnospiraceae_NK4A136_group* is an anaerobic and spore-forming bacteria that belongs to the *Lachnospiraceae* family (Vacca et al., 2020). It is one of the primary genera present in the intestine of mice and is regarded as a potentially beneficial bacterium (Yan et al., 2023). *Lachnospiraceae_NK4A136_group* is inversely associated with various metabolic diseases and chronic inflammation and can produce SCFAs by fermenting dietary polysaccharides (Truax et al., 2018; Ma et al., 2020). It exhibits anti-inflammation effects in mice with obesity (Hu et al., 2019) and a potential anti-colitis activity (Zhang et al., 2020). The abundance of *Lachnospiraceae_NK4A136_group* decreased in mice with ulcerative colitis after stimulation with dextran sodium sulphate (Wu et al., 2022). Moreover, it exhibited a negative correlation with shoulder fat, AST and liver weight (Wu et al., 2023) while demonstrated a significant association with enhanced IMBF (Ma et al., 2020). *Desulfovibrio* belongs to sulphate-reducing bacteria and is present as resident commensal bacteria within the human gastrointestinal tract, which can produce H_2_S as a terminal by-product of their metabolic activity (Singh et al., 2023). While H_2_S exhibits important beneficial effects on gastrointestinal integrity and ulcer healing (Magierowski et al., 2015). *Faecalibaculum* is widely found in the gastrointestinal tract and produces anti-inflammatory molecules, such as acetic acid and butyric acid, to maintain intestinal health and stability (Guo Q. et al., 2023; Song et al., 2023; Yi et al., 2023). In addition, a recent study has shown that *Faecalibaculum* is negatively correlated with liver function indicators (ALT, AST, ALP, LDH), systemic inflammation indicators (LPS, TNF-α, IL-6, IL-1β) and positively correlated with colon index (colon length and PAS + goblet cell/crypt) (Zhang et al., 2023). In brief, the dominant flora of SDC treatment group could hinder IMBF in various ways, nevertheless, the change of microbiota caused by the addition of GYY4137 had a beneficial effect on the maintenance of IMBF.

This research also has its limitations. First, the unconformity in the change of tight junction protein expression between cell and animal study may be caused by different research models and the insufficient treatment time for the Caco-2 monolayer, which needs further study in the future. Second, the authors did not analyze the ultrastructure of TJs. Third, the molecular mechanism of IMBF impairment caused by SDC has not been thoroughly studied. Some studies have discovered that it may be related to the activation of MAPK and NF-*κ*B pathway, the modulation of miRNA and the gut microbiota-farnesoid X receptor axis (Stenman et al., 2012; Chen et al., 2016; Rawat et al., 2020; Xu et al., 2021; Liu et al., 2022; Petito-da-Silva et al., 2023). The next research goal is to explore molecular mechanisms. Finally, if the differences in the microbiome between different groups can be further verified through fecal microbiota transplantation, the experimental results will be more reliable.

## 5 Conclusion

In conclusion, this study demonstrates that GYY4137 could ameliorate SDC-induced chronic intestinal barrier injury both *in vitro* and *in vivo* for the first time. GYY4137 may play a protective role by inhibiting the MLCK-P-MLC2 pathway, suppressing decreased expression level of TJs, anti-apoptosis and maintaining the homeostasis of the microbiome. The study findings provide an essential foundation for using GYY4137 to treat deoxycholic acid–related intestinal barrier damage, providing a novel target for preventing and treating high–fat diet-related intestinal diseases.

## Data Availability

The data presented in the study are deposited in the NCBI repository, accession number PRJNA1151985 and PRJNA1151967.
