# Supplementary material for: GYY4137, as a slow-releasing H2S donor, ameliorates sodium deoxycholate–induced chronic intestinal barrier injury and gut microbiota dysbiosis
Source: Front Pharmacol. 2024 Oct 22;15:1476407. doi: 10.3389/fphar.2024.1476407 (PMC11539038; doi:10.3389/fphar.2024.1476407)
Supplement: Supplementary file 5 [file DataSheet1.docx]

Supplementary Material

# Supplementary Figures


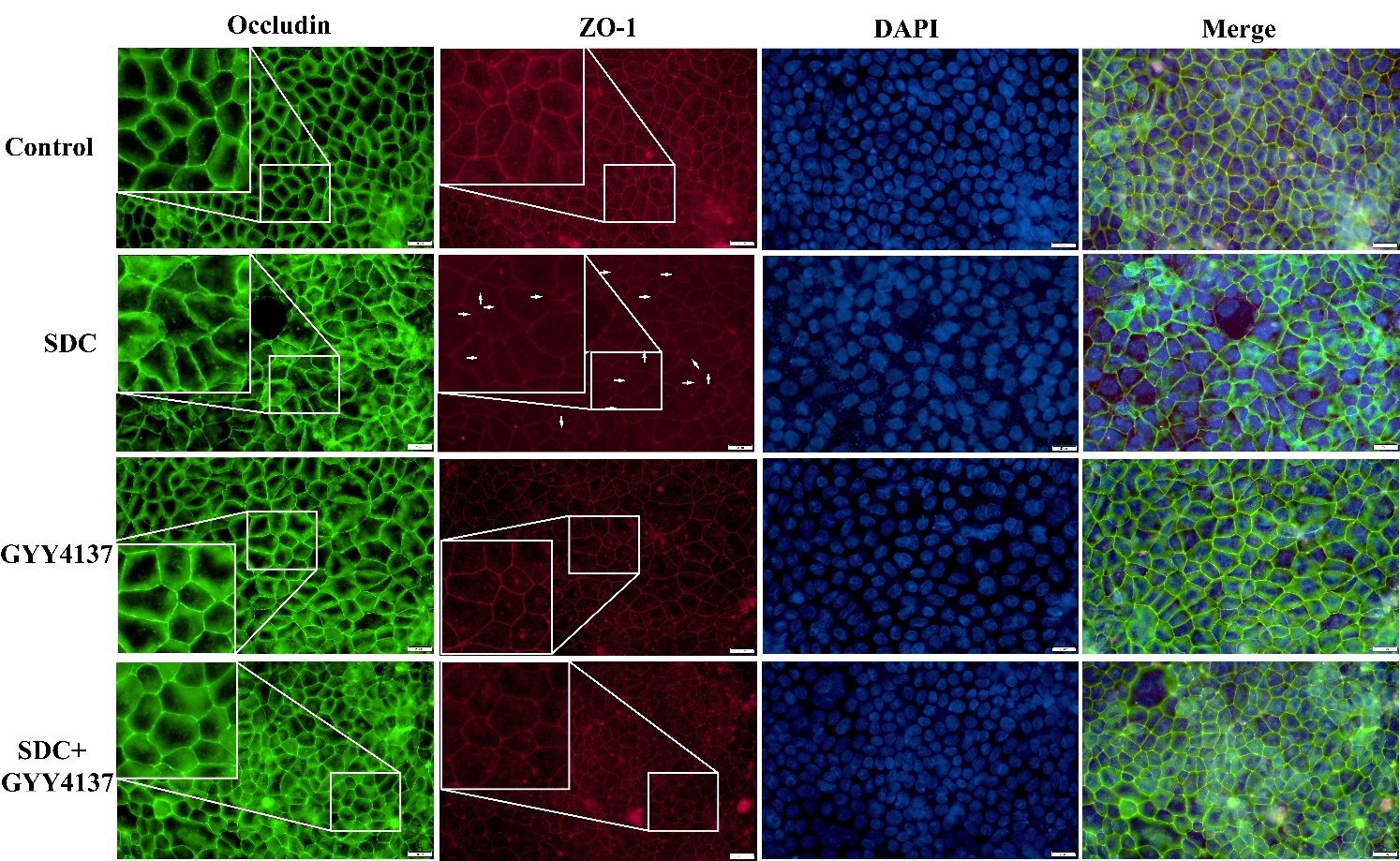
**Supplementary Figure 1.** Occludin and ZO-1 were stained by immunofluorescence in Caco-2 monolayers.


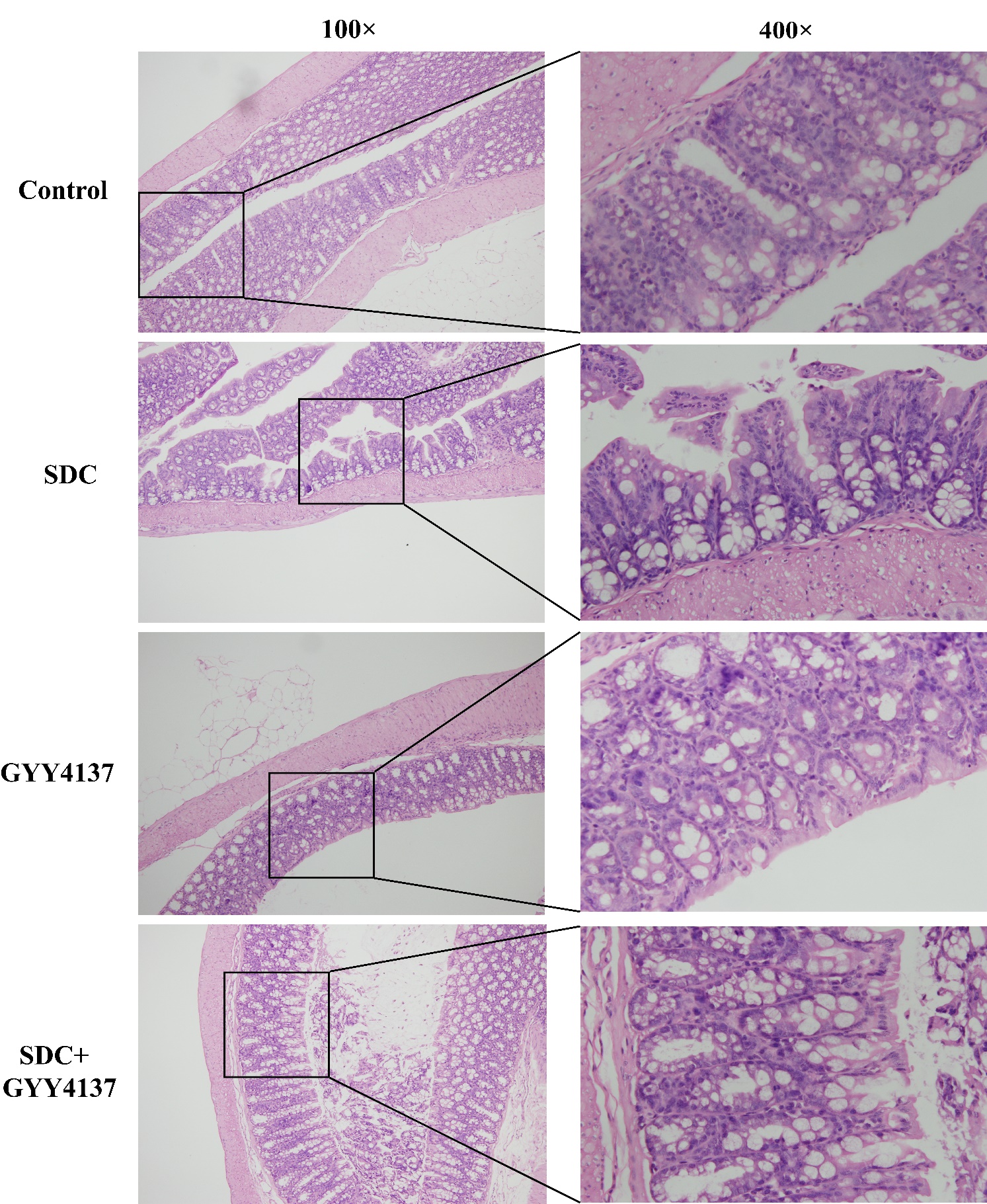


**Supplementary Figure 2.** GYY4137 remarkably attenuated the mucosal damage in mice treated with SDC.


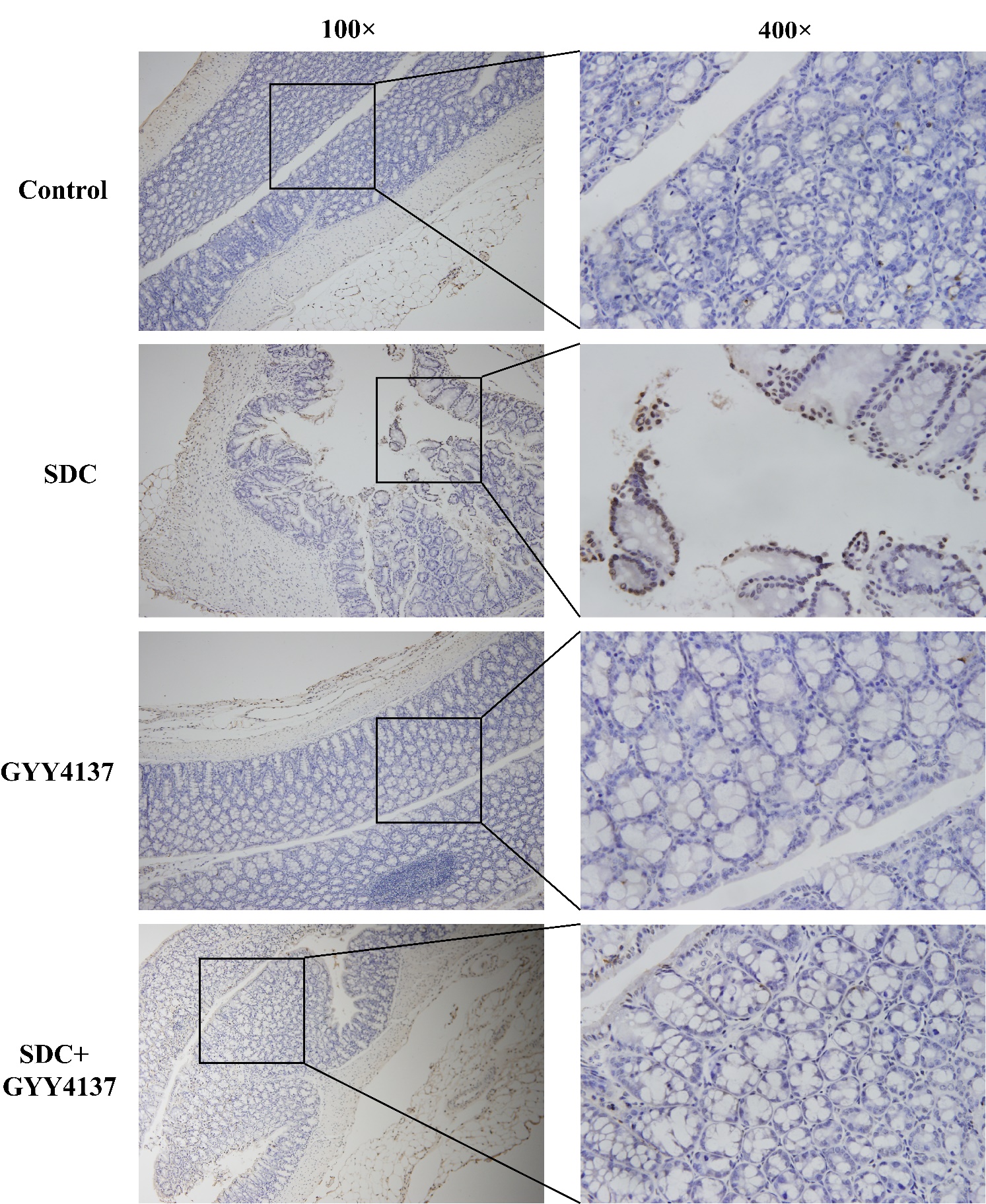


**Supplementary Figure 3.** Representative TUNEL images for cell apoptosis (brown signals) and GYY4137 significantly decreased the increased level of apoptosis in the colon epithelium in mice fed with SDC.


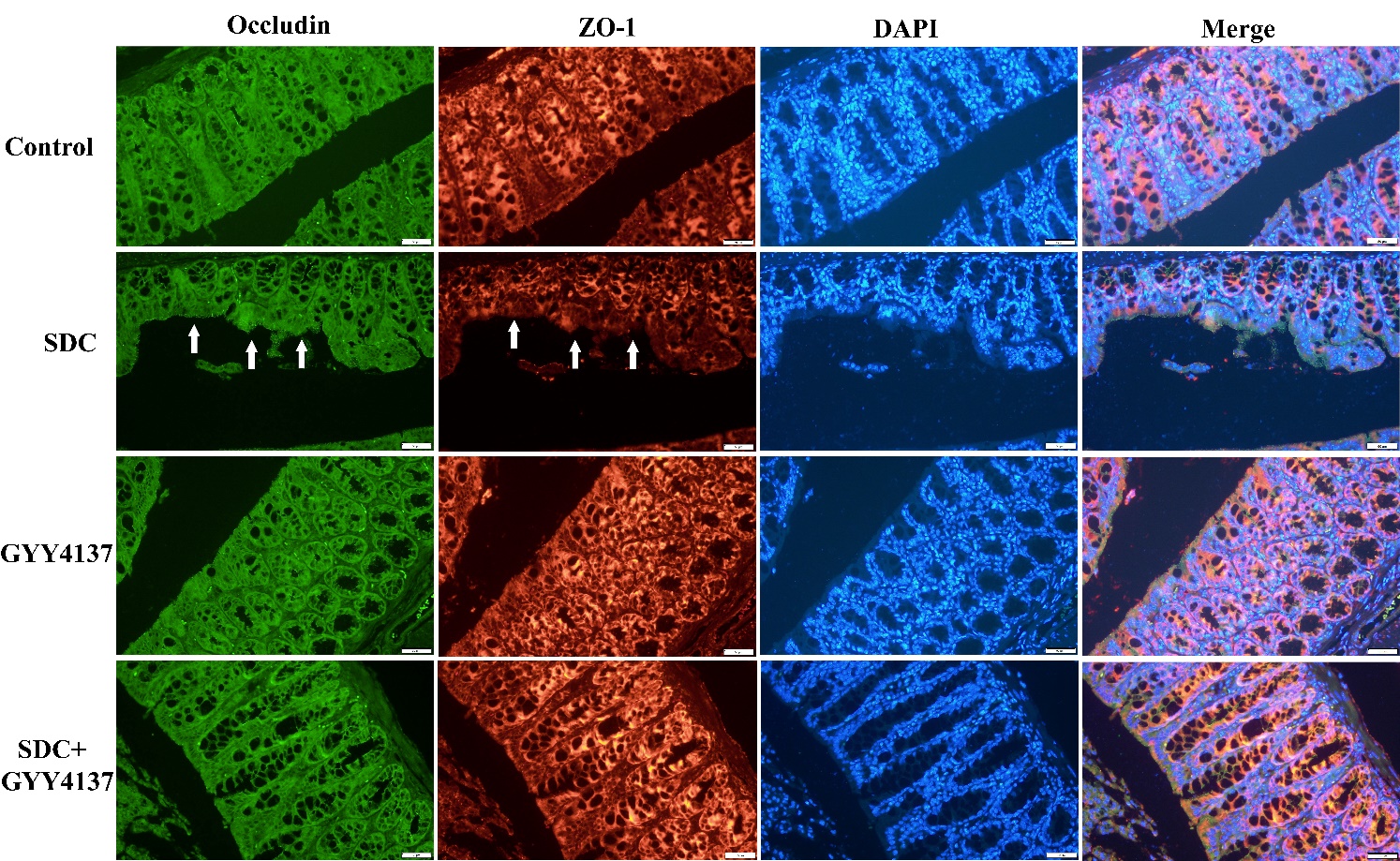


**Supplementary Figure 4.** Occludin and ZO-1 were stained by immunofluorescence. GYY4137 inhibited their decreased staining intensity and the damaged mucosal integrity in mice treated with SDC (White arrows were added to direct against distinct changes).
